# Method for QCM Resonator Device Equivalent Circuit Parameter Extraction and Electrode Quality Assessment

**DOI:** 10.3390/mi12091086

**Published:** 2021-09-09

**Authors:** Dong Liu, Xiaoting Xiao, Ziqiao Tang, Qiao Chen, Haoran Li, Xiaoxiong Wang, Yan Yan

**Affiliations:** 1 School of Automation Engineering, University of Electronic Science and Technology of China, Chengdu 611731, China; dong.liu@uestc.edu.cn (D.L.); qiaochen@std.uestc.edu.cn (Q.C.); horn_lee@std.uestc.edu.cn (H.L.); yan.y@uestc.edu.cn (Y.Y.); 2 School of Electrical Engineering and Information, Southwest Petroleum University, Chengdu 610599, China; xiaoting.xiao@swpu.edu.cn; 3 Wuhan Hi-Trusty Electronics Co., LTD, Wuhan 432400, China; xiaoxiongwng@gmail.com

**Keywords:** quartz crystal microbalance, piezoelectric resonator, piezoelectric generator, electrode quality assessment, equivalent circuit parameter extraction

## Abstract

Quartz crystal microbalance (QCM) resonators are used in a wide range of sensors. Current QCM resonators achieve a simultaneous measurement of multiple physical quantities by analyzing lumped-element equivalent parameters, which are obtained via the introduction of external devices. This introduction of external devices will probably increase measurement error. To realize the measurement of multiple physical quantities while eliminating the measurement error caused by external devices, this paper proposes a measurement method for the lumped-element equivalent parameters of QCM resonators without the need for extra external devices. Accordingly, a numerical method for solving nonlinear equations with fewer data points required and a higher accuracy was adopted. A standard crystal resonator parameter extraction experiment is described. The extracted parameters were consistent with the nominal parameters, which confirms the accuracy of this method. Furthermore, six QCM resonator device samples with different electrode diameters and materials were produced and used in the parameter measurement experiment. The linear relationship between the electrode material conductivity and motional resistance *R*_1_ is discussed. The ability of this method to characterize the electrode material and to detect the rust status of the electrode is also demonstrated. These abilities support the potential utility of the proposed method for an electrode quality assessment of piezoelectric devices.

## 1. Introduction

Quartz crystal microbalances (QCM), as microelectromechanical devices, have been widely used in sensing applications, including humidity [1,2,3,4], liquid [5], and gas sensors [6]. Based on the adsorption effect of polymers, DNA, or proteins [7], QCM sensors have great application prospects in the field of biosensors, e.g., quality inspection in the biopharmaceutical industry [8] and applications in artificial olfaction [9], among others. Biosensors are primarily used in a liquid environment, and QCM loading has an inhomogeneous structure [10]. Under these conditions, QCM has an effect not only on the mass [11] but also on the viscosity [12]. This phenomenon is manifested in the complexity behind the frequency shift interpretation when QCM is used in liquid applications [11]. Commercial QCM sensors only provide limited information such as resonance frequency; however, the equivalent parameters provide a higher observation dimension, which is convenient for dissociating multiphysical coupling effects, realizing multiphysical sensors [13], and improving sensitivity [14,15,16]. Additionally, the equivalent parameters can be used to evaluate the performance of devices. Therefore, it is necessary to extract equivalent parameters for the purpose of aligning the sensor design with their practical applications [17].

Moreover, QCM equivalent circuit parameter extraction has applications in other piezoelectric resonators. It was reported that QCM resonators, AlN piezoelectric resonators [18,19], thin-film microelectromechanical system (MEMS) resonators [20], and piezoceramic transducers [21] have similar equivalent circuit models. Therefore, the equivalent circuit parameter extraction method presented here can also be used as a potential approach to the characterization of piezoelectric resonators.

QCM sensors can be described with a Mason equivalent circuit [22,23]. After several mathematical simplifications, the equivalent circuit can be described as a Butterworth–Van Dyke (BVD) equivalent circuit [24]. Many researchers have contributed to the QCM equivalent parameter measurement of BVD equivalent circuits. Casteleiro-Roca et al. [25] obtained the equivalent parameters by a series inductance and resistance. Liu et al. [26] measured the equivalent parameters by a series standard capacitance. Due to the connection of external electronic components, optimization and/or tuning is required in both of the above methods. Consequently, in situ equivalent parameter measurement methods without the need for external devices were developed. Yao et al. [14] measured the impedance of QCM sensors with an impedance analyzer and determined the equivalent parameters by fitting. Gugliandolo et al. [17] extracted the equivalent parameters via scattering (S-) parameter measurements coupled with a Lorentzian fitting; however, this fitting method requires more data points, which increases the probability of noise interference.

This paper presents a method to measure the equivalent parameters based on the phase–frequency curve of insertion loss (S21) of the resonator device. This method adopts an analytical solution instead of fitting. It extracts only four points of the phase–frequency curve, which are near the resonance or antiresonance frequency. The slope of a phase–frequency curve is greater when the frequency point is near the resonance or antiresonance frequency and is not sensitive to phase jitter caused by noise. Therefore, this method has a higher signal-to-noise ratio (SNR) than fitting-based methods. The combination of higher SNR and reliance on fewer data points increases the accuracy of the parameter measurement, which is reflected by the small root mean square error (RMSE) value between the measurement curve and the inversion curve.

The accuracy of the proposed method was verified via a parameter measurement of a standard crystal. Additionally, QCM resonator devices with different electrode diameters and materials were analyzed to demonstrate the ability of this method to determine the electrode radius and to evaluate the electrode rust status. Finally, the capability of this method to evaluate the electrical performance of a resonator is demonstrated.

## 2. Methodology and Simulation Validation

### 2.1. Methodology

The derivation of the proposed method is provided below and then verified by simulation.

**Figure 1:**
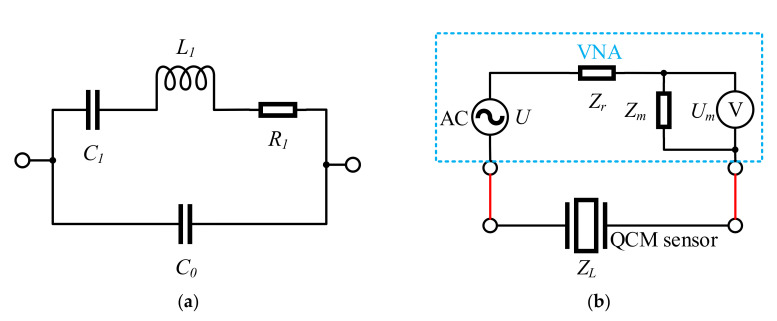
Schematic diagrams of the equivalent circuit of the QCM sensor and the measuring circuit: (**a**) Butterworth–Van Dyke (BVD) equivalent circuit model of the QCM sensor; (**b**) vector network analyzer (VNA) measuring circuit.

The equivalent circuit model of the QCM device is shown in Figure 1a and is referred to as the Butterworth–Van Dyke (BVD) model [27]. It contains four parameters: static capacitance *C*_0_, motional capacitance *C_1_*, motional inductance *L*_1_, and motional resistance *R*_1_. The schematic diagram of the measurement circuit is shown in Figure 1b, where *Z_r_* is the internal resistance, *Z_m_* is the measurement resistance, and *Z_L_* is the load impedance. In this case, *Z_L_* is the impedance of the network in Figure 1a.

The ratio of the vector voltage on the measuring resistance to the vector voltage of the AC signal source is given by the following:

(1) $$G\left(f\right)=\frac{U_{m}}{U}=\frac{Z_{m}}{Z_{L}+Z_{r}+Z_{m}}$$

where *U_m_* is the vector voltage on the measurement resistance and *U* is the vector voltage of the AC signal source. Then, the phase of the forward transmission coefficient *S*_21_ is as follows [26]:

(2) $$\mathrm{Phase}\left(S_{21}\right)=\frac{180}{\pi }\mathrm{atan}\left(\frac{\mathrm{imag}\left(G\left(f\right)\right)}{\mathrm{real}\left(G\left(f\right)\right)}\right)$$

The magnitude of the forward transmission coefficient is:

(3) $$\mathrm{Mag}\left(S_{21}\right)=\mathrm{abs}\left(G\left(f\right)\right)$$

The zero-phase frequency of the network in Figure 1a is [26]:

(4) $$\omega_{\pm }=\sqrt{\left(\frac{1}{L_{1}C_{1}}+\frac{1}{2L_{1}C_{0}}-\frac{R_{1}{}^{2}}{2L_{1}{}^{2}}\right)\pm \sqrt{{\left(\frac{1}{L_{1}C_{1}}+\frac{1}{2L_{1}C_{0}}-\frac{R_{1}{}^{2}}{2L_{1}{}^{2}}\right)}^{2}-\frac{1}{L_{1}{}^{2}C_{1}{}^{2}}\left(1+\frac{C_{1}}{C_{0}}\right)}}$$

The resonance angular frequency is:

(5) $$\omega_{r}=\sqrt{\left(\frac{1}{L_{1}C_{1}}+\frac{1}{2L_{1}C_{0}}-\frac{R_{1}{}^{2}}{2L_{1}{}^{2}}\right)-\sqrt{{\left(\frac{1}{L_{1}C_{1}}+\frac{1}{2L_{1}C_{0}}-\frac{R_{1}{}^{2}}{2L_{1}{}^{2}}\right)}^{2}-\frac{1}{L_{1}{}^{2}C_{1}{}^{2}}\left(1+\frac{C_{1}}{C_{0}}\right)}}$$

The antiresonance angular frequency is:

(6) $$\omega_{a}=\sqrt{\left(\frac{1}{L_{1}C_{1}}+\frac{1}{2L_{1}C_{0}}-\frac{R_{1}{}^{2}}{2L_{1}{}^{2}}\right)+\sqrt{{\left(\frac{1}{L_{1}C_{1}}+\frac{1}{2L_{1}C_{0}}-\frac{R_{1}{}^{2}}{2L_{1}{}^{2}}\right)}^{2}-\frac{1}{L_{1}{}^{2}C_{1}{}^{2}}\left(1+\frac{C_{1}}{C_{0}}\right)}}$$

It could be found that the resonance angular frequency *ω_r_* and the antiresonance angular frequency *ω_a_* have the relationship given by the following:

(7) $$\frac{\omega_{a}{}^{2}+\omega_{r}{}^{2}}{2}=\frac{1}{L_{1}C_{1}}+\frac{1}{2L_{1}C_{0}}-\frac{R_{1}{}^{2}}{2L_{1}{}^{2}}$$

(8) $$\omega_{r}{}^{2}\omega_{a}{}^{2}=\frac{1}{L_{1}{}^{2}C_{1}{}^{2}}\left(1+\frac{C_{1}}{C_{0}}\right)$$

It could then be found that Equations (7) and (8) contain equivalent parameters. Therefore, a method exists for the retrieval of BVD parameters from the two frequency points *ω_r_* and *ω_a_*. However, the four unknown parameters correspond to two input frequencies, and the equations have innumerable solutions. Consequently, two other equations with BVD parameters as independent variables needed to be constructed. The derivative equations of the phase–frequency curve at the resonance frequency and the antiresonance frequency were, thus, added to obtain four nonlinear quaternion equations.

In practice, the derivative can be approximated as the slope of the curve. Therefore, the two additional equations required were constructed as follows:

(9) $$\Delta_{1}={\left.\frac{\partial \left(\mathrm{Phase}\left(S_{21}\right)\right)}{\partial f}\right|}_{f=f_{r}}$$

(10) $$\Delta_{2}{\left.=\frac{\partial \left(\mathrm{Phase}\left(S_{21}\right)\right)}{\partial f}\right|}_{f=f_{a}}$$

where *f_r_* is resonance frequency and *f_r_* = *ω_r_/*2*/π*; *f_a_* is antiresonance frequency and *f_a_* = *ω_a_/*2*/π*; Δ_1_ and Δ_2_ are the slopes of the phase–frequency curve at the resonance and antiresonance frequency, respectively.

Due to the automatic impedance matching of the instrument, the internal resistance *Z_r_* changes with changes in the load resistance *Z_L_*. Therefore, the internal resistance *Z_r_* must be corrected. In the proposed method, the maximum value of the magnitude-frequency curve was selected to correct *Z_r_* [28]. The additional equation is:

(11) $$\mathrm{Max}\left[\mathrm{Mag}\left(S_{21}\right)\right]=\mathrm{abs}\left(G\left(f_{s}\right)\right)$$

where Max[Mag(*S*_21_)] is the maximum value of the measured magnitude–frequency curve and *f_s_* is the frequency corresponding to the maximum point.

Thus, the nonlinear equations are given as follows [28]:

(12) $$\left\{\begin{array}{c} \frac{\omega_{a}{}^{2}+\omega_{r}{}^{2}}{2}=\frac{1}{L_{1}C_{1}}+\frac{1}{2L_{1}C_{0}}-\frac{R_{1}{}^{2}}{2L_{1}{}^{2}}\\ \omega_{r}{}^{2}\omega_{a}{}^{2}=\frac{1}{L_{1}{}^{2}C_{1}{}^{2}}\left(1+\frac{C_{1}}{C_{0}}\right)\\ \Delta_{1}={\left.\frac{\partial \left(\mathrm{Phase}\left(S_{21}\right)\right)}{\partial f}\right|}_{f=f_{r}}\\ \Delta_{2}{\left.=\frac{\partial \left(\mathrm{Phase}\left(S_{21}\right)\right)}{\partial f}\right|}_{f=f_{a}}\\ \max\left[\mathrm{Mag}\left(S_{21}\right)\right]=\mathrm{abs}\left(G\left(f_{s}\right)\right) \end{array}\right.$$

In Formula (12), the feature parameters extracted from the measurement curve are presented on the left and the parameters to be calculated are shown on the right. Similar to the finite element method, the four-dimensional space, including *R*_1_, *L*_1_, *C*_1_, and *C*_0_, was constructed first. Subsequently, the four parameters were solved by the variable step search method [29].

### 2.2. Validation by ADS Simulation

**Figure 2:**
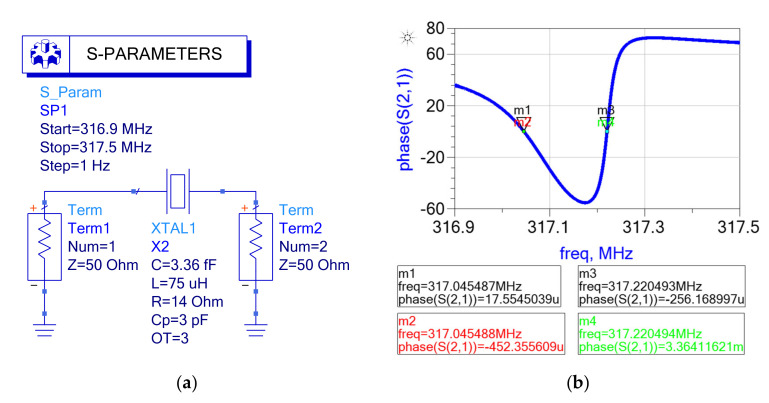
Simulation schematic diagram and result: (**a**) schematic diagram; (**b**) phase–frequency curve of quartz crystal ADS simulation.

Advanced Design System (ADS, Keysight Technologies Ltd., Santa Rosa, CA, USA) software was used for the simulation of the BVD model, and the frequency response of the circuit was obtained via the S-parameter simulation module. The start frequency of the S-parameters was 316.9 MHz, the stop frequency was 317.5 MHz, and the frequency spacing of scanning was 1 Hz. The XTAL1 module was used to characterize the QCM resonator, and Term 1 and Term 2 characterized the internal resistance and measurement resistance, respectively, both of which were 50 Ω. The simulation schematic and its generated phase–frequency curve are shown in Figure 2. The simulation parameters were derived from an actual resonator [30] and were *R*_1_ = 14.00 Ω, *L*_1_ *=* 75.00 uH*, C*_1_ *=* 3.36 fF, and *C*_0_ *=* 3.00 pF.

As shown in Figure 2a, the internal resistance *Z_r_* (Term 1) and measurement resistance *Z_m_* (Term 2) were 50 Ω. As the internal resistance was invariable in the simulation model, only the phase–frequency curve was needed to calculate the equivalent parameters. The equivalent parameter calculation steps were as follows:

**Step 1, figure acquisition**. The phase–frequency curve for the Advanced Design System (ADS) simulation shown in Figure 2b was obtained.

**Step 2, feature parameter extraction.** Feature parameters were extracted from the phase–frequency curve. In Figure 2b, the two points closest to the resonance frequency were m1 (317,045,487 Hz, 17.55 udeg.) and m2 (317,045,488 Hz, −452.36 mdeg.), the two points closest to the antiresonance frequency were m3 (317,220,493 Hz, −256.17 udeg.) and m4 (317,220,494 Hz, 3.36 mdeg.).

**Step 3,input parameter calculation.** The resonance frequency and slope were calculated from points m1 and m2: the resonance frequency was *f_r_* = 317045487.03 Hz and the slope was *Δ_1_* = −4.69 × 10^−4^ deg./Hz. The antiresonance frequency and slope were calculated from points m3 and m4: the antiresonance frequency was *f_a_* = 317220493.07 Hz and the slope was *Δ_2_* = 3.62 × 10^−2^ deg./Hz.

**Step 4,solution to the nonlinear equations.** The input parameter values (*f_r_*, *f_a_*, Δ_1_, Δ_2_, *Z_r_*, and *Z_m_*) were substituted into the nonlinear equations in Formula (12) to solve for the output parameters.

**Step 5, results.** The output parameters calculated according to the proposed method were *R*_1_ = 14.00 Ω, *L*_1_ = 74.99 uH, *C*_1_ = 3.36 fF, and *C*_0_ = 2.99 pF. Compared with the setting parameters, the maximum error was 0.0067%.

The simulation results verified the high accuracy of the equivalent parameters measured by the proposed method. In Section 3, the accuracy of this method was verified using a standard crystal experiment. The potential of this method to evaluate the crystal properties was demonstrated by the experimental results.

## 3. Experimental Platform and Materials

To validate our simulation results, an experimental platform was set up and included a vector network analyzer (VNA), a conditioning circuit, and a software algorithm derived from the proposed method.

A total of seven resonator devices—one standard crystal resonator device and six QCM resonator devices—were used in the testing. All of the QCM resonator devices were manufactured by Wuhan Hi-Trusty Electronics Co., Ltd. Three of them differed by their electrode radius diameters, and the rest varied in their electrode materials.

### 3.1. Experimental Platform

**Figure 3:**
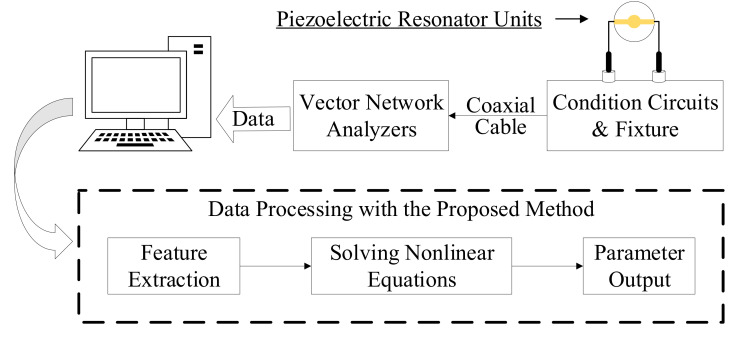
Schematic diagram of the experiment.

A block diagram of the experiment is shown in Figure 3. The resonator was connected to the vector network analyzer (VNA) through the conditioning circuit and fixture. The device communicates with the host computer by USB and transmits the measured S-parameter data to the computer. The measured data were processed by the proposed method, and the equivalent parameters were calculated.

### 3.2. Material

The testing materials included one standard crystal resonator device and six QCM resonator devices.

#### 3.2.1. Standard Crystal Resonator Device

In order to verify the accuracy of this method, the standard resonator parameters were measured by the proposed method and were compared with those measured by the supplier using large-scale instruments.

The standard resonator was provided by Hebei BOWEI Integrated Circuits Co., Ltd. (Shijiazhuang, China). The parameters that were measured by the producer using professional equipment were *R*_1_ = 69.78 Ω, *L*_1_ = 1407.29 mH, *C*_1_ = 0.18 pF, and *C*_0_ = 2.11 pF.

#### 3.2.2. QCM Resonator Devices with Different Electrode Diameters and Materials

As a microelectromechanical device, the electrode shape or material of a QCM resonator greatly affects its electrical properties. Thus, the relationship between the electrode radius, the electrode material, and equivalent parameters was investigated. The equivalent parameters of QCM resonators with three different electrode diameters and three types of electrode materials were measured, and the relationships between static capacitance and the electrode diameter and between motional resistance and the electrode material were analyzed. The experimental results demonstrate the potential of this method in the evaluation of QCM properties.

**Figure 4:**
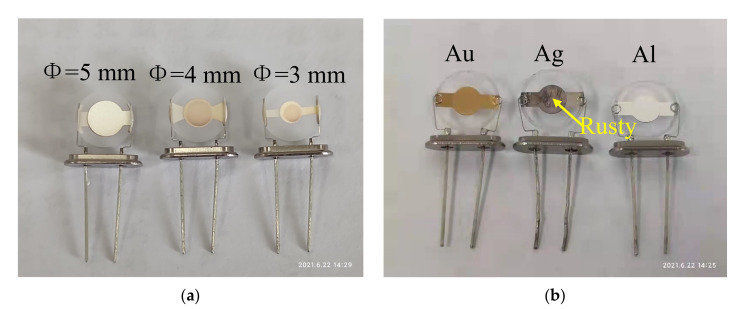
QCM resonator devices with different electrode diameters or materials: (**a**) QCM resonator devices with different electrode diameters; (**b**) QCM resonator devices with different electrode materials.

Figure 4a shows the three QCM resonator devices with different electrode diameters. Their diameters were Φ = 5 mm, Φ = 4 mm, and Φ = 3 mm, and the electrode material of all three was silver.

Figure 4b shows the three QCM resonator devices with different electrode materials. The electrodes were composed of gold (Au), silver (Ag), and aluminum (Al), and all had a diameter of Φ = 4 mm. In industrial applications, the aging of electrodes due to oxidation is a common phenomenon in QCM devices and occurs most commonly in silver electrodes. As such, a QCM device with an aging silver electrode, including some rusting, was chosen for the experiments, thereby allowing for the potential of the proposed method for the evaluation of electrode aging to be determined.

## 4. Results and Discussion

The experimental results are divided into three parts: (1) the measurement of the standard resonator device parameters, (2) the equivalent parameter measurement of the QCM resonator devices with different electrode diameters, and (3) the equivalent parameter measurement of the QCM resonator devices with different electrode materials.

This study also discusses the quantitative characterization of a QCM resonator through: (1) the linear relationship between the electrode area, the static capacitance *C*_0_ and motional capacitance *C*_1_, and (2) the linear relationship between electrode material conductivity and motional resistance *R*_1_.

Based on this discussion, the ability of the proposed method to detect electrode rust status was also demonstrated.

### 4.1. Measurement of Standard Resonator Device Parameters

**Figure 5:**
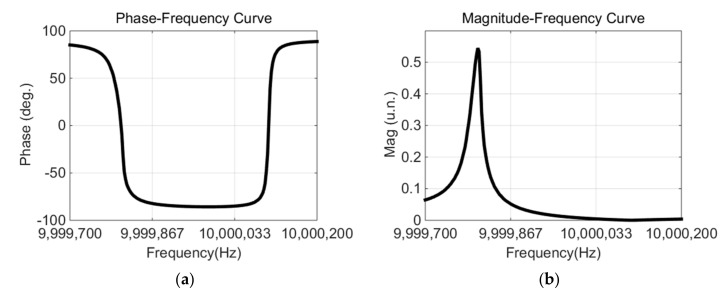
Measured curve and inversion curve of standard resonator: (**a**) phase–frequency curve; (**b**) magnitude–frequency curve.

The phase–frequency curve and magnitude–frequency curve measured by VNA are shown in Figure 5a,b.

Through the proposed method, the equivalent circuit parameters of this standard resonator were found to be *R*_1_ = 70.59 Ω, *L*_1_ = 1447.23 mH, *C*_1_ = 0.17 pF, and *C*_0_ = 2.87 pF. A comparison between the parameters measured by this method and those provided by the supplier is presented in Table 1.

The parameters measured by this method were basically consistent with those measured by a supplier’s large-scale instruments, and the discrepancies were mainly due to *C*_0_. This was due to the difference in fixtures between the supplier’s measurement system and our measurement system [26]. When *C*_0_ was removed, the maximum error of the other parameters was less than 5%.

### 4.2. Equivalent Parameter Measurement of QCM Resonator Devices with Different Electrode Diameters

**Figure 6:**
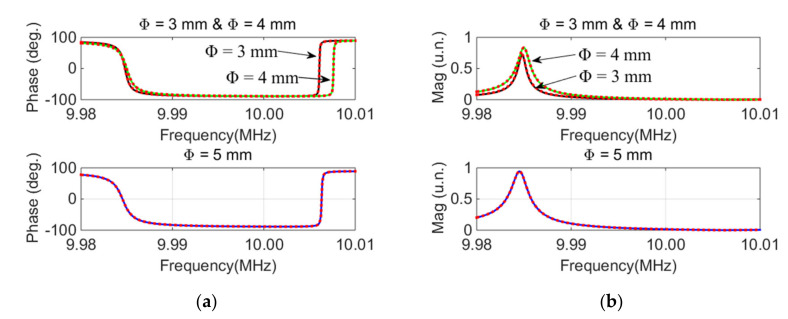
Measured curve and inversion curve of QCM resonator devices with different electrode diameters: (**a**) Phase-frequency curve; (**b**) Magnitude-frequency curve.

The phase–frequency and magnitude–frequency curves of QCM devices with different electrode diameters are presented in Figure 6 (solid line).

The dotted lines show the inversed curves of the equivalent circuit parameters presented in Table 2. The inversion curves were obtained by substituting the parameters presented in Table 2 into Formulas (2) and (3). It can be seen that the measurement curve and the inversion curve were highly consistent, which indicates the accuracy of the measurement parameters. The RMSE values between the measured and inversion phase–frequency curves for devices with 3 mm, 4 mm, and 5 mm diameter electrodes were 0.52, 0.31, and 0.16, respectively. The RMSE values were low, which further validates the accuracy of the measured parameters.

**Figure 7:**
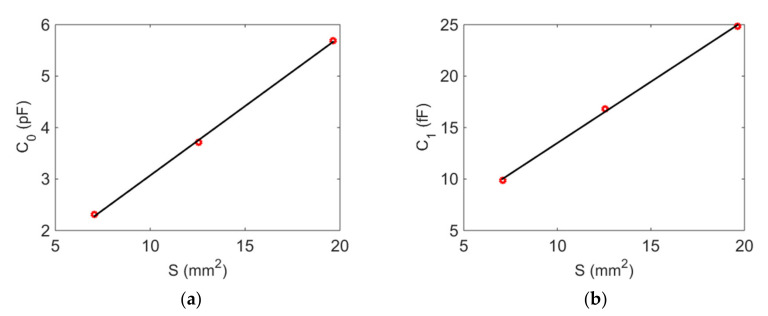
The linear relationship between electrode area and both *C*_0_ and *C*_1_: (**a**) *S* vs. *C*_0_; (**b**) *S* vs. *C*_1_.

Through a further analysis of the data in Table 2, it was found that the capacitance diameter exerted an influence on all four parameters but had little influence on the capacitance ratio *r*, defined as *r = C*_0_*/C*_1_ [27]. This was because both *C*_0_ and *C*_1_ were positively proportional to the electrode area [27]. The relationship between the electrode area and both *C*_0_ and *C*_1_ can be seen in Figure 7: *C*_0_ and *C*_1_ had a highly linear relationship with the electrode area, which verifies the accuracy of the calculation parameters. Additionally, the feasibility of this method for the detection of the electrode area is shown.

### 4.3. Equivalent Parameter Measurement of QCM Resonator Devices with Different Electrode Materials

**Figure 8:**
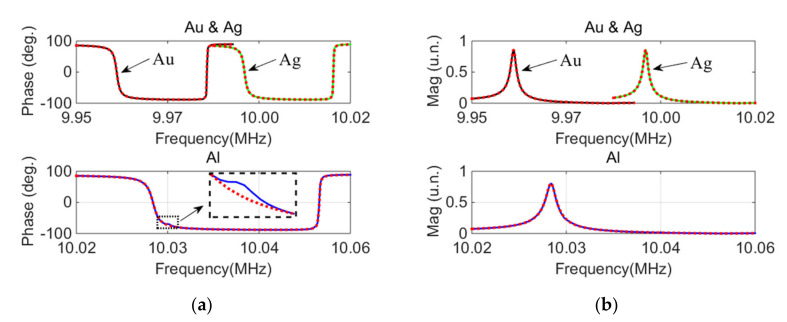
Measured curve and inversion curve of QCM resonator devices with different electrode materials: (**a**) phase–frequency curve; (**b**) magnitude–frequency curve.

The phase–frequency curve and magnitude–frequency curve of QCM devices with different electrode materials are shown in Figure 8 (solid line). The materials tested were Au, Ag, and Al.

The equivalent parameters of QCM devices with different electrode materials were measured by the proposed method, as shown in Table 3.

The dotted lines show the inversed curves of the equivalent circuit parameters presented in Table 3. The RMSE values between the measured and inversion phase–frequency curves for devices with Au, Ag, and Al electrodes were 1.08, 0.57, and 0.81, respectively.

It can be found from Figure 8a that the measured phase–frequency curve of the Al electrode had a large jitter at 10.03 MHz. This jitter was caused by the inevitable noise of the measuring environment. The black dotted box shown in Figure 8a is an enlarged view of the jitter of the measured curve. The blue solid line is the curve as measured by the instrument. The red dotted line is the curve of parameter inversion. It can be seen that the dotted line was smoother; if the curve was fitted using these unsmooth data points, then the fitting error would increase.

Comparatively, the proposed method only used four points from the phase–frequency curve rather than all of them—as required for the fitting-based method. These four points were close to the resonance and antiresonance frequency. The slope of the curve was larger at the resonance and antiresonance frequency (as shown in Figure 8a). Thus, the phase jitter had a smaller effect on the smoothness of the curve. Consequently, the phase–frequency curve inverted by the parameters measured by the proposed method was highly consistent with the measured curve, except for in the area of jitter.

The above experiments demonstrated the advantage of the proposed method by reducing the influence of the phase jitter on the parameter measurement. This could explain why the RMSE of the curves inverted by the parameters measured by the proposed method was as low as 0.81.

Through a further analysis of the data in Table 3, the differences between the *C*_0_ values for the three devices with different electrode materials were found to be minimal, with a standard deviation of 6.24%. This was likely due to the consistent electrode diameter for the three devices (Φ = 4 mm).

It can be seen from Table 3 that the greatest influence of electrode material was on the motional resistance *R*_1_. This may be due to the different conductivity of the electrode materials. The conductivity of the electrode materials and their motional resistance *R*_1_ are presented in Table 4.

**Figure 9:**
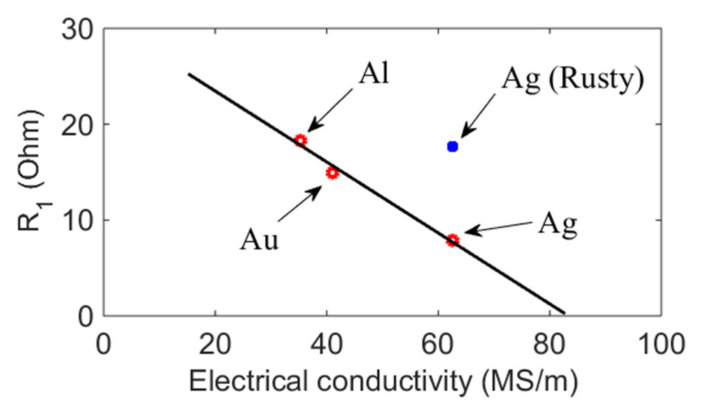
The linear relationship between electrode material conductivity and motional resistance *R*_1_.

To demonstrate the relationship between the electrode material conductivity and motional resistance *R*_1_, the data from Table 4 were plotted in Figure 9. The conductivity of the electrode material had a linear relationship with motional resistance; however, the device with a rusty silver electrode (blue dot) did not exhibit this same relationship.

The above experiments demonstrated that the proposed method could be used to evaluate the rust status of QCM resonator electrodes, which suggests a potential application for these devices in other fields.

## 5. Conclusions

A method for the extraction of the equivalent parameters of the BVD model of QCM resonator devices was presented in this paper. The proposed method requires neither external electronic components nor optimization and/or tuning. Moreover, the numerical method of nonlinear equations was adopted in place of the fitting method. Special feature points were selected and reduced the influence of the phase jitter noise on the measurement of parameters, which resulted in RMSE values as small as 0.16 between the curve inverted from the parameters and the actual measurement curve. Compared with the supplier’s instrument-based testing results, the measuring error was less than 5%. The typical tolerance of precision commercial inductors was ±5% [25]. In 2017, Liu’s method utilized a connected external capacitor to reduce the measuring error to ±4.5% [26]. By removing the need to connect external devices, the error of the proposed method was further reduced to ±2.5%.

Some potential applications of this method were given. QCM resonator devices with three electrode diameters and three types of electrode material were manufactured. The linear relationship between the static capacitance and the electrode area, and the linear relationship between the conductivity of the electrode material and the motional resistance, were demonstrated via parameter measurement experiments. We also demonstrated the potential of this method to detect electrode rusting.

This method was suitable for the BVD model and has the potential to be applied to other piezoelectric resonators, such as AlN piezoelectric resonators, thin-film MEMS resonators, and piezoceramic transducers. Therefore, our study provides a potential approach for the quality assessment of other piezoceramic resonators.

For the convenience of communication, we provide the URL of the program used to calculate the parameters from the curve measured by VNA. URL: https://pan.baidu.com/s/1KrLw3-Kgips6ct04gaA76g. Password is vtky.

## Figures and Tables

**Table 1:** Comparison between the parameters measured by the proposed method and those provided by the supplier.

Parameters	Proposed Method	Supplier Measurement
*R*_1_*(*Ω)	70.59	69.78
*L*_1_ (mH)	1447.23	1407.29
*C*_1_ (pF)	0.17	0.18
*C*_0_ (pF)	2.87	2.11

**Table 2:** Equivalent parameters of QCM resonator devices with different electrode diameters.

Φ (mm)	3	4	5
*R*_1_ (Ω)	12.42	7.87	6.63
*L*_1_ (mH)	25.77	15.10	10.22
*C*_1_ (pF)	9.86	16.82	24.86
C_0_ (pF)	2.31	3.71	5.69
*r*	4.29	4.53	4.36

**Table 3:** Equivalent parameters of QCM resonator devices with different electrode materials.

Electrode Material	Au	Ag	Al
*R*_1_ (Ω)	15.00	17.70	18.33
L_1_ (mH)	15.31	15.61	16.10
*C*_1_ (pF)	16.67	16.25	15.64
*C*_0_ (pF)	3.60	3.57	3.69

**Table 4:** Conductivity of the electrode materials and their motional resistance.

Electrode Material	Ag	Au	Al	Ag (Rusty)
*R*_1_ (Ω)	7.87	15.00	18.33	17.70
Conductivity (MS/m)	62.50	41.00	35.34	62.50

## Data Availability

Data available in a publicly accessible repository that does not issue DOIs. Publicly available datasets were analyzed in this study. This data can be found here: https://pan.baidu.com/s/1KrLw3-Kgips6ct04gaA76g. Password is vtky. The other data presented in this study are available on request from the corresponding author.
